# Health Tract, No. 79

**Published:** 1865

**Authors:** 


HALL'S JOURNAL OF HEALTH, Vol. 12, No. 5

### HEALTH TRACT, No. 79.

### SUMMERINGS.

1. In going to the country to spend your summer, leave business behind, but take with you your entire stock of patience, courtesy, self-respect, and religion. Go as plain "John Smith, gentleman."

2. If you have the first claim to being well-bred, you will be the last person in the world to volunteer any information on the subject. If it must be told, let it be by your conduct; let your entire deportment prove that you are a lady or a gentleman.

3. Do not profess that you "know" Mr. Astor, Mr. Grinnell, Mr. Minturn, or other distinguished citizens, when your entire knowledge consists in their having been pointed out to you on the street.

4. Avoid claiming acquaintance with this or that family of note, when you only happen to have spoken to them on a rail-car or steamboat, or in some purely business transaction. An enterprising individual once claimed that he knew a distinguished judge very well. On inquiry it was found that the said judge had once sent him to the penitentiary!

5. If you have the first mite of common-sense, and really go to the country for recreation, enjoyment, and health, leave your best and second-best clothing at home; take only your common wardrobe, and but a small part of that; not only that the persons you "stop with" may feel more easy, but that you may feel freer yourself to scale fences, climb trees, scramble up mountain-sides, wade across creeks, penetrate forest tangles, and jump Jim Crow generally.

6. Never turn up your nose at any thing at the table; if you have the slightest disposition to do so, you may be sure it is a pug, and isn't long enough to turn. If you don't like a thing, let it alone; eat nothing, and by the next meal you may be glad to get any thing.

7. Remember that in going to the country a sensible man's object is neither to dress nor eat, chiefly, but to obtain mental repose, pure air, and unrestrained exercise.

8. Endeavor to conform, without apparent effort, to the arrangements of the family with whom you board, and to the manners and customs of the people around you, as far as they do not compromise your principles of good morals and good taste.

9. Be cheerful, be kind, be considerate, be accommodating.

10. Do not obtrude your political or religious sentiments.

11. Shun argument and controversy on any and all subjects.

12. Let your courtesy come out naturally; and if religious, don't be a Pharisee.

